# Stratification of Health Professional Education and Its Funding Disparities: Evidence From China During the Period of 1998–2017

**DOI:** 10.3389/fpubh.2021.800163

**Published:** 2022-01-18

**Authors:** Wenjuan Gao, Wenzhuo Li, Yue Zang, Yuxin Zhong, Hongbin Wu

**Affiliations:** ^1^Institute of Higher Education, Beihang University, Beijing, China; ^2^Education Section, Aerospace Center Hospital/Peking University Aerospace School of Clinical Medicine, Beijing, China; ^3^Institute of Medical Education, Peking University, Beijing, China; ^4^Graduate School of Education, Peking University, Beijing, China; ^5^National Center for Health Professions Education Development, Peking University, Beijing, China

**Keywords:** financing, health professional education, stratification, health professional institutions, funding disparities

## Abstract

**Background:** The finance of health professional education (HPE) is of immense importance for effective and sustainable health systems, yet relevant empirical research was scarce due to the lack of financial data. The study aimed to bridge the gap by presenting the scenario of finance for health professional institutions (HPIs) of different tiers in China and exploring how the stratification of institutions affected their funding disparities.

**Methods:** The study employed data collected from the Ministry of Education in China, and selected the HPIs mainly based on the World Directory of Medical Schools. The funding levels and disparities of China's HPIs during the period (1998–2017) were analyzed with descriptive statistics, and the indicators of funding per institution and funding per student were both considered. The average funding in HPIs was presented by tiers, and the Gini coefficient and Theil index were employed to describe the differences in financing among HPIs over the span.

**Results:** The study found that the number of HPIs has kept growing over the past two decades, with both the funding per institution and the funding per student increasing steadily. Specifically, the average funding per institution of the three tiers increased by 31.5 times, 13.4 times, and 10.5 times separately, with the first-tier universities having an absolute advantage compared to lower tiers. As for the financing disparities among HPIs, the Gini coefficient of the funding per institution maintained to be over 0.5, with the third-tier institutions scoring the highest, while the Gini coefficient of the funding per student all ranged approximately from 0.2 to 0.3. Through the decomposition of the inequalities measured by the Theil index, the share of the between-tier difference in per-institution funding grew from 29.7 in 1998 to 77.9% in 2017.

**Conclusions:** The funding disparities between tiers of HPIs in China gradually became more accentuated, with the top-tier institutions taking up the largest share. Although the stratified development in HPE has posed a challenge to the unified quality assurance of medical personnel training, it may also be regarded as an effective pathway for developing countries like China to achieve stable development in health professional education.

## Introduction

Funding serves as the basis for the development of health professional education (HPE), which ensures normal and effective teaching activities in health professional institutions (HPIs). In particular, public financing should be allocated properly so as to improve efficiency in the national contexts (1). The extant research on higher education finance focuses mainly on fiscal inequality (2), performance-based funding policies (3), the impacts of financial incentives measures on student educational outcomes (4), as well as the determinants of government funding (5), etc. However, related fields have not formed a very clear outline of funding input in HPE and the financial situation of HPIs worldwide. Some studies have examined the financial investment in American medical schools (6, 7), while some others discussed the financing in postgraduate HPE (8), as well as the funding in residents (9), yet there is relatively little empirical research on the finance of HPE compared with the ample exploration on the financing of tertiary education (10). One of the important reasons, as stated in the report published in *The Lancet* in 2010, is that there is a lack of financial data in HPE (1).

A joint report *Health Professionals for a New Century* published by the global commission on education of health professionals for the 21st century noted that the HPE reform in the coming 100 years will require a series of enabling actions, such as enhancement of investment level of HPE, which is a key measure to advance the quality and equity of HPE (1). In the report, a macro estimation approach was adopted by examining the funding of higher education as well as the proportion to HPE, and results showed that the estimated funding per medical student was $122 000 worldwide in the year 2008. The estimated value in Western Europe was similar to those in North America ($497 000 and $400 000, respectively), but was much lower in China ($14 000, 1/35 of North America, and 1/28 of Western Europe) (1).

The funding income of HPIs is directly related to the operational status of the HPIs and will influence the quality of medical students' training. In view of these realities, the commission's initiative is that each country and agency should consider doubling its investments in HPE over the next 5 years as an indispensable contributor for effective and sustainable health systems (1).

## Stratification of the Health Professional Education in China

### Horizontal Differentiation of Chinese Health Professional Institutions

The Ministry of Education (MOE) in China has categorized higher education institutions into comprehensive universities and 11 types of single-subject colleges, including medicine and pharmacy colleges, natural sciences and technology colleges (11). In the early 21st century, some highly competitive colleges of medicine and pharmacy merged into comprehensive universities in China (12); thus, HPE has been offered not only by colleges of medicine and pharmacy, but also by comprehensive universities which include medicine-related departments. In 2017, China had 631 comprehensive universities and 199 colleges of medicine and pharmacy, including both the undergraduate and vocational colleges (13).

China has cultivated the largest quantity of medical students worldwide, though its number of HPIs is not the largest (1). In 2017, Chinese higher education institutions enrolled 7.6 million students in total, with 0.5 million students being admitted into HPIs, accounting for 6.6% of the total (13). In general, Chinese health professional institutions consist of 11 disciplines, namely clinical medicine, nursing, dental medicine, traditional Chinese medicine (TCM), public health and preventive medicine, the combination of modern medicine with TCM, pharmaceutical science, traditional Chinese pharmaceutical science, forensic medicine, allied health and basic medicine (11). Taking clinical medicine as an example, its enrollment took up a large proportion of the total to medicine, but the proportion has been declining year by year, from 63.2 in 1998 to 31.6% in 2012, and then remained at around 30% in recent years. In addition, undergraduate enrollment accounts for about 50% of the total clinical enrollment, while the ratio of master's and doctoral degrees has increased with time, indicating the growing demand for higher educational attainment (14).

### Vertical Hierarchy of Higher Education Institutions in China

A series of schemes have been consecutively launched by the Chinese government including *Project 211, Project 985*, and the *Double First-Class* program over years in order to improve the education in elite universities and to facilitate the development of tertiary education (15). Specifically, *Project 211* was initiated in 1995 with the purpose of setting a priority to improve the education of leading universities and to enhance their research standards, and the selected 115 prestigious universities have received 70% of the national research funding from 1995 to 2008 (16). In 1998, *Project 985* was launched to build world-class universities and 39 elite universities were chosen to be equivalent to the US Ivy League, which have received substantial public funding and policy support (17). In the year 2015, the *Double First-Class* program, known as the continuation of the two previous projects with a broader geographical spread, was inaugurated aiming to build an outstanding higher education system with first-class universities and first-class disciplines. Altogether 137 universities out of over 2000 universities have been selected, which formed the three selectivity tiers of institutions. The tier 1 comprises 42 universities (including all 39 of the *Project 985* institutions, and three additional universities from the former *Project 211*), which were regarded to have the potential to reach world-class standards. The tier 2 consists of 95 universities (mainly the former *Project 211* institutions) which were identified to have strengths in particular disciplines with a solid foundation for development. The tier 3 refers to other non-*Project 211* universities (18, 19).

The distribution of HPIs follows the overall layout of higher education in China (20). In terms of financing allocation, the funding of health professional institutions mainly comes from their competent authorities. HPIs of the top tiers in China are directly affiliated to the MOE or other central ministries, thus their financial resources are mainly from the central government. The second-tier HPI are institutions of higher education with key medical disciplines, which are directly affiliated to the National Health Commission (NHC), and jointly established by the NHC or the National Administration of Traditional Chinese Medicine, the MOE, as well as the local governments. The third-tier HPIs are mainly affiliated to the local ministries, so the local governments provide them the main financial support. There exists a mutual influence between the development situation of HPIs and their financial resources to some extent. Sufficient funding plays a basic and supportive role in ensuring the development of HPIs, while well-developed HPIs, in return, may attract further funding sources.

The extant literature has examined the cost and financial demand of general higher education, but little research has focused specifically on HPE that may function very differently. Relevant studies were either case studies of certain institutions or general analyses based on personal experience, which can hardly provide an overall picture of the national funding situations and disparities among HPIs in China. Therefore, this paper aimed to bridge the gap by analyzing the changing trends of the funding among HPIs of different tiers from 1998 to 2017, and exploring how the stratification of institutions affects the funding disparities across tiers.

## Methods

### Data Sources

This study employed data collected from the MOE in China, which encompasses the funding status, student number, and basic information of institutions of higher education nationwide. It should be noted that the statistical calibers of financial revenue and expenditure in China are quite different from the US. The primary sources of funds for public higher educational institutions in China are fiscal appropriation, tuition, fees and scientific research income, operating income, donations and grants, funding for institutional infrastructure, as well as other funding from miscellaneous sources. The financial expenditure includes salaries, wages and employee benefits, purchased goods and services, capital outlay, and other expenditures. Since hospitals are organized and operated as legal entities independent from HPIs, the expense in medical services was not included in the data. HPIs in our study mainly refer to colleges and universities that offer the clinical medicine programs, and usually these universities also have programs of other medical disciplines. HPIs in our study have been selected through three steps: (1) A total of 160 HPIs in the mainland of China have been screened based on the World Directory of Medical Schools (WDMS) provided by the World Federation for Medical Education (WFME) (21). (2) Our research excluded 22 private HPIs that were listed in the WDMS, given that the statistical calibers of financial revenue and expenditure are quite different between the public and private HPIs, and China's higher educational system is dominated by public institutions. (3) We checked the relevant data year by year from 1998 to 2017 considering that the annual lists of HPIs varied since the clinical medicine programs had been established in different years. Thus, all the HPIs in our paper are public institutions offering degree programs, and in accordance with the *Double First-Class* initiative, this paper posits a classification of HPIs into three tiers. The tier 1 HPIs are key universities that have the potential to reach world-class standards; the tier 2 HPIs are institutions with key disciplines; and the tier 3 HPIs are other institutions. We used the 2017 US dollar constant price as the measurement standard by adjusting the funding data based on China's consumer price index (CPI) in 2017, and then converting Chinese Yuan to US Dollar (1 USD ≈ 6.752 CNY) according to the exchange rate in 2017 (22).

### Data Analysis: Gini Coefficient and Theil Index

The study aimed to present the funding levels and disparities of different tiers of China's HPIs with descriptive statistics. We first compared the means of funding in HPIs when analyzing the finance distributions as well as changes across years, and the indicators of both funding per institution and funding per student were considered in our analysis. Furthermore, the Gini coefficient and Theil index were employed to describe the differences in finance among HPIs over the years. All the statistical analyses were performed using Stata version 15.1. The calculation of both Gini coefficient and Theil index of funding per institution and funding per student comes from the study by Cowell (23). The Gini coefficient, derived from the Lorenz curve framework, ranges between 0 and 1, indicating the income distribution from perfectly equitable to perfectly inequitable (24, 25). According to the United Nation standard, a Gini coefficient of <0.2 denotes absolute equality, while values of 0.2–0.3 represent relative equality; values of 0.3–0.4 stand for relatively reasonable inequality; values of 0.4–0.5 mean relatively big inequality; values over 0.5 represent severe inequality. Gini coefficients can be calculated with the equation 1. *n* refers to the total number of samples in a certain year; *W*_*i*_ stands for the percentage of funding of group 1 to *i* in proportion to that of all HPIs.


(1) $$\begin{array}{r} Gini=1-\frac{1}{n}\left(2\sum_{i\;=\;1}^{n-1}W_{i}+1\right) \end{array}$$

Theil index, another measure of inequality, can decompose the differences from within-group and between-group separately, which ranges between 0 and infinity, with greater values indicating increasing levels of inequality (26–28). Theil Index can be measured through the equation 2. *n* refers to the total number of the sample, which are divided into *k* groups, and group *k* was shown as *g*_*k*_, while the number of the group *k* was *n*_*k*_; *y*_*i*_ and *y*_*k*_ stand for the share of individual *i* and the share of group *k*, respectively. Our study decomposed the Theil index by the three tiers of HPIs (see equation 3 and 4). *P*_*b*_ refers to the share of between-tier difference. *P*_*w*_ refers to the share of within-tier difference. *T*_*b*_ and *T*_*w*_ represent the differences between and within tiers, used for calculating the share of intra-group and inter-group contribution to the total difference.


(2) $$\begin{array}{l} \begin{array}{ccc} Theil & = & \frac{1}{n}{\sum^{\text{​}}}^{\text{​}}{}_{i\;=\;1}^{n}\frac{y_{i}}{\bar{y}}\ln(\frac{y_{i}}{\bar{y}})={\sum^{\text{​}}}^{\text{​}}{}_{k=1}^{k}y_{k}\ln\frac{y_{k}}{\frac{n_{k}}{n}} \end{array}\\ \;\begin{array}{c} +{\sum^{\text{​}}}_{k=1}^{k}y_{k}({\sum^{\text{​}}}_{i\in g_{k}}\frac{y_{i}}{y_{k}}\ln\frac{\frac{y_{i}}{y_{k}}}{\frac{1}{n_{k}}})=T_{b}+T_{w} \end{array} \end{array}$$

(3) $$\begin{array}{rcl} P_{b} & = & \frac{T_{b}}{Theil}\times 100\% \end{array}$$

(4) $$\begin{array}{rcl} P_{w} & = & \frac{T_{w}}{Theil}\times 100\% \end{array}$$

## Results

### Distribution of Financial Resources in Health Professional Institutions

Table 1 reports the number of HPIs and the average funding per institution by tiers between 1998 and 2017 in China. In general, the number of health professional institutions kept growing over the span. In the year 1998, there were altogether 94 HPIs, with 17 top-tier institutions, 9 s-tier institutions, and 68 third-tier institutions. By 2017, the number had increased to 152 HPIs in China, and the quantities for the three tiers were 23, 16, and 113, respectively. As for the per institution funding, it has risen by 17.5 times from $14.2 million in 1998 to $262.3 million in 2017 on average. Specifically, the average funding per institution of the three tiers increased by 31.5 times, 13.4 times, and 10.5 times separately. Until 2017, the average funding per institution for the three tiers has reached up to $ 982.2, $ 223.4, and $ 113.1 million, respectively; and it is obvious that the first-tier institutions have received the largest share of financial resources.

**Table 1:** Average funding per institution by tiers of health professional institutions: 1998–2017.

**Year**	**Total**		**Tier 1**		**Tier 2**		**Tier 3**	
	***N***	**Mean (US$ in millions)**	***N***	**Mean (US$ in millions)**	***N***	**Mean (US$ in millions)**	***N***	**Mean (US$ in millions)**
1998	94	14.2	17	30.2	9	15.5	68	9.8
1999	96	21.7	19	55.0	9	19.8	68	12.1
2000	101	44.9	19	156.4	9	31.8	73	15.6
2001	106	65.0	20	229.7	11	40.4	75	20.2
2002	113	70.6	20	260.5	12	46.3	81	23.5
2003	115	75.1	20	269.2	12	53.4	83	27.7
2004	119	77.8	20	268.0	12	57.3	87	33.7
2005	121	88.3	20	304.2	12	75.4	89	38.6
2006	125	98.7	20	358.4	13	78.2	92	41.9
2007	126	114.6	20	414.6	13	97.4	93	48.7
2008	126	120.9	20	435.9	13	103.7	93	51.7
2009	127	136.2	20	502.7	13	104.8	94	58.1
2010	132	156.8	20	614.6	13	125.9	99	64.3
2011	133	184.2	20	702.4	13	154.3	100	79.9
2012	136	190.7	20	715.7	14	162.6	102	84.8
2013	137	194.3	20	727.6	14	177.1	103	88.8
2014	140	235.8	21	885.3	14	224.8	105	102.2
2015	141	230.7	21	867.4	14	214.9	106	101.6
2016	149	235.8	21	928.8	16	225.4	112	101.2
2017	152	262.3	23	982.2	16	223.4	113	113.1

*The funding per institution across years has been adjusted based on China's CPI index in the year 2017, and the Chinese RMB was converted to USD (1 USD ≈ 6.752 CNY) according to the exchange rate in 2017*.

Table 2 presents the average funding per student of HPIs in China during the period. The funding per student on average nationwide grew from $2,695 in 1998 to $6,175 in 2017, though there had been a slight decline in the early 21st century possibly affected by the expansion of institutions. The average funding per student in all three tiers have experienced similar upward trends to $13,147, $5,804, and $4,728 separately until 2017, with the top-tier universities had absolute advantage compared to the other two tiers. This suggested that training a student at top-tier health professional institutions in China usually costs up to ~$65,700 during the five years of undergraduate study. In addition, the average annual growth rate in the first-tier institutions reached 7.11%, which was significantly higher than its counterparts in tier 2 (4.5%) and tier 3 (3.4%) institutions.

**Table 2:** Average funding per student by tiers of health professional institutions: 1998–2017.

**Year**	**Total (US$ in thousands)**	**Tier 1 (US$ in thousands)**	**Tier 2 (US$ in thousands)**	**Tier 3 (US$ in thousands)**
1998	2.695	3.560	2.513	2.492
1999	3.083	4.491	2.627	2.727
2000	3.274	5.674	2.601	2.692
2001	3.248	5.900	2.704	2.547
2002	3.125	6.035	2.548	2.434
2003	3.105	5.441	3.036	2.508
2004	3.027	4.989	3.108	2.531
2005	3.283	5.323	3.754	2.736
2006	3.372	6.104	3.451	2.732
2007	3.658	6.689	3.184	3.034
2008	3.743	6.893	3.218	3.099
2009	3.899	7.711	3.260	3.129
2010	4.384	9.209	3.821	3.439
2011	5.057	10.190	4.597	4.045
2012	5.017	9.981	4.812	4.008
2013	4.984	9.803	5.244	3.977
2014	5.734	11.825	6.304	4.395
2015	5.538	11.499	5.666	4.295
2016	5.619	12.275	5.871	4.277
2017	6.175	13.147	5.804	4.728

*The funding per institution across years has been adjusted based on China's CPI index in the year 2017, and the Chinese RMB was converted to USD (1 USD ≈ 6.752 CNY) according to the exchange rate in 2017*.

### Funding Disparities Across Health Professional Institutions in China

**Figure 1:**
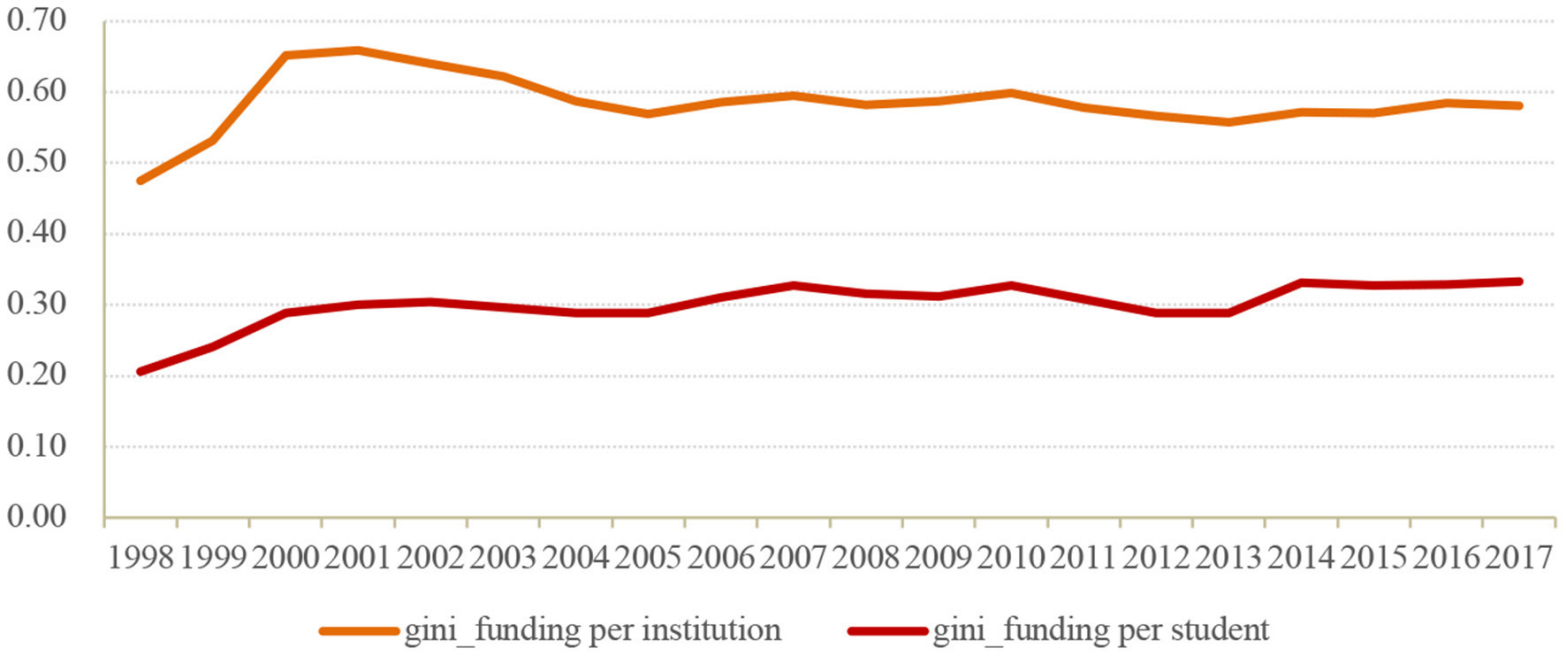
Gini coefficient of funding per institution and funding per student among HPIs in China.

Figure 1 presents the Gini coefficients of the funding per institution and the funding per student in HPIs over years. Overall, both the Gini coefficient of the funding per institution and the funding per student had increased steadily from 1998 to 2000, after which they experienced a slow decline before they started to remain stable since 2005. The Gini coefficient of the funding per institution maintained to be over 0.5, while the Gini coefficient of the funding per student ranged approximately from 0.2 to 0.3, indicating that the distribution of financial resources among institutions were quite inequal, while the funding was relatively equally allocated among students in general.

**Figure 2:**
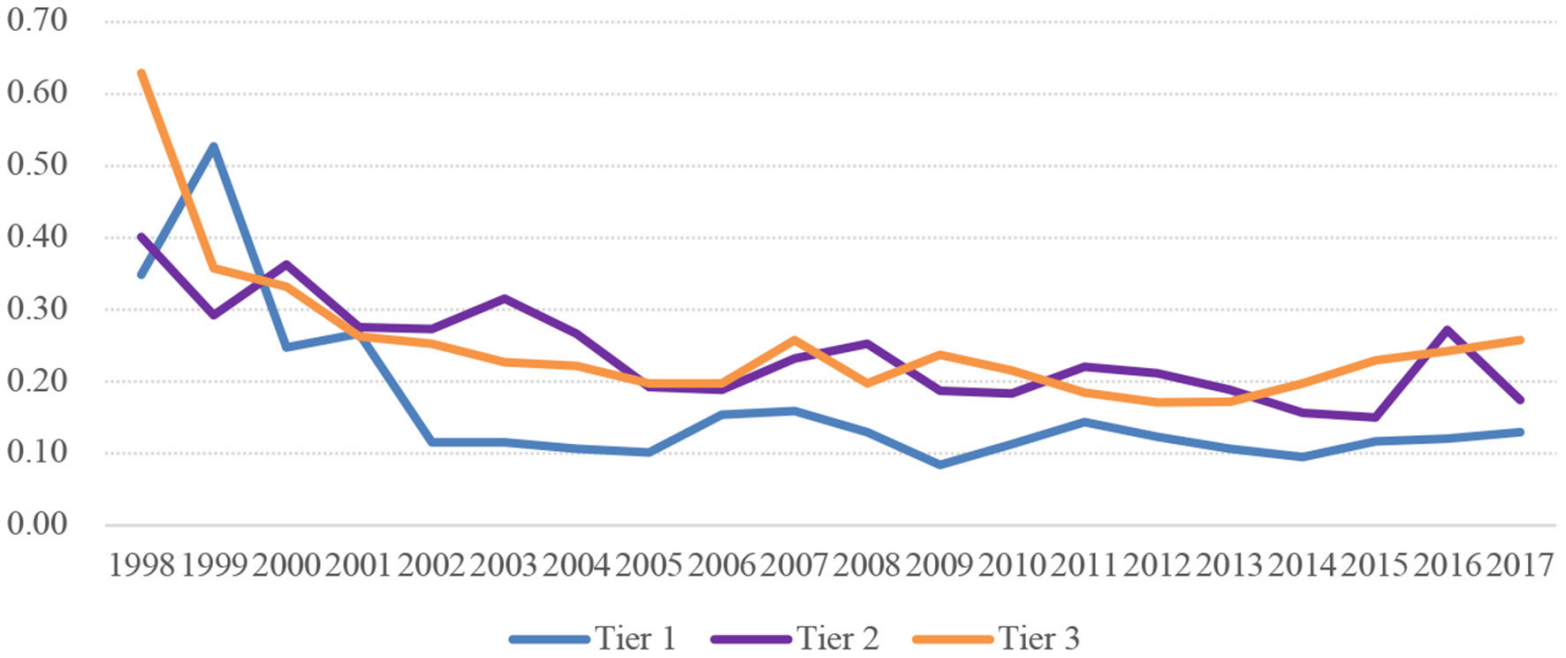
Gini coefficient of funding per institution in HPIs of three tiers in China.

**Figure 3:**
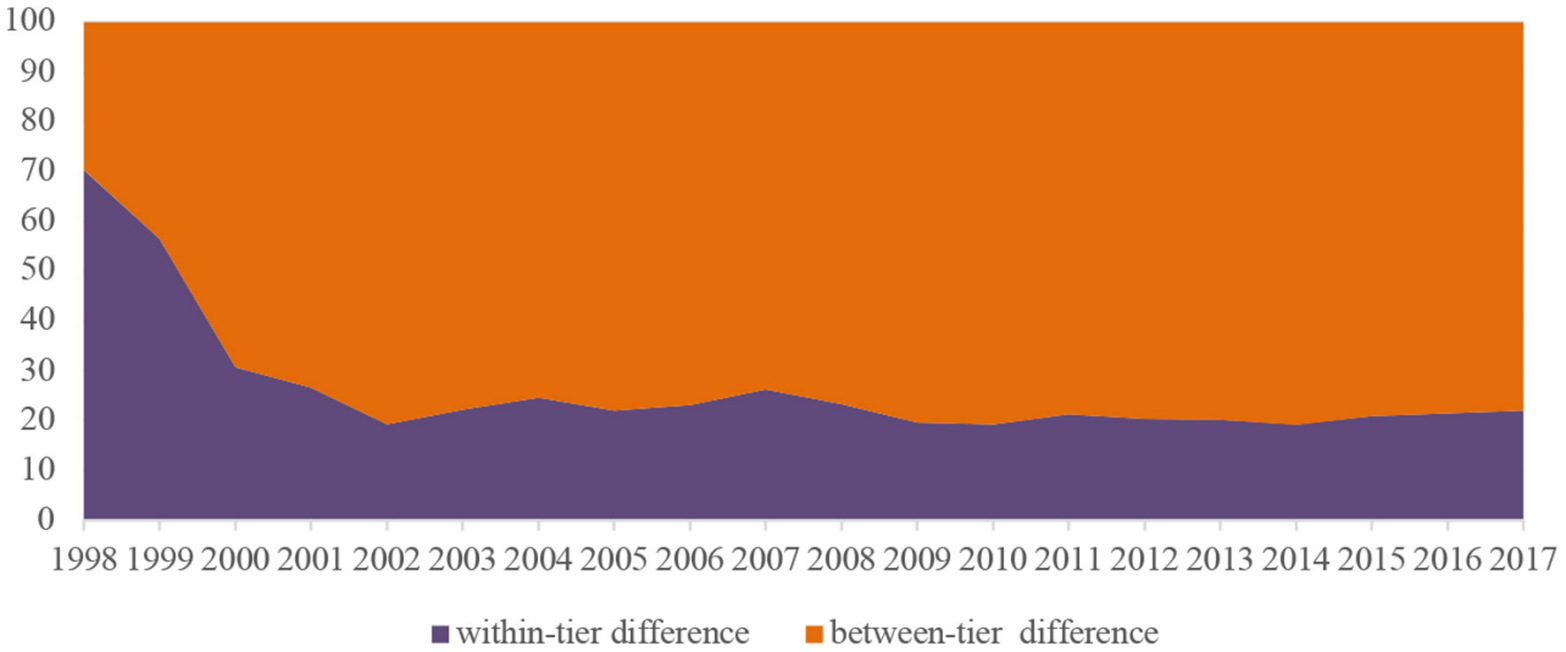
Within-tier and between-tier differences in funding per institution among HPIs in China.

Figures 2, 3 show the disparities in per institution funding in HPIs across tiers. According to Figure 2, the Gini coefficients in HPIs of the three tiers all experienced an overall decline regarding the funding per institution over years. In general, the financial resources were more equitably distributed within the top tiers of institutions, while the funding allocation among the lower-tier colleges and universities appeared to be more unfair. Figure 3 further distinguishes the differences between the three tiers of the HPIs in question and the differences within each tier of institutions. The share of within-tier difference declined from 70.3% in 1998 to 22.1% in 2017, indicating that the differences in per institution funding were more distinct within each tier from 1998 to 1999, and since the expansion of higher education, the funding disparities were increasingly noticeable between different tiers after 2000.

**Figure 4:**
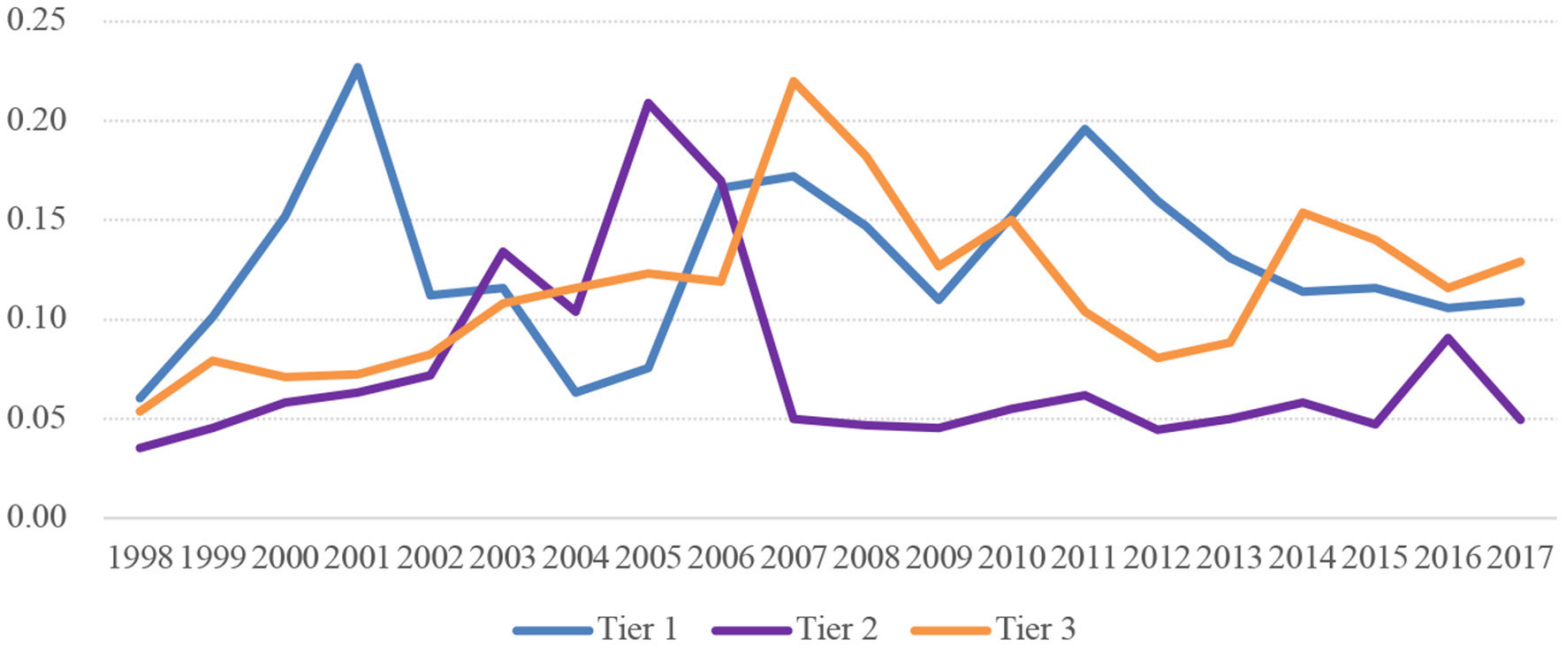
Gini coefficient of funding per student in HPIs of three tiers in China.

**Figure 5:**
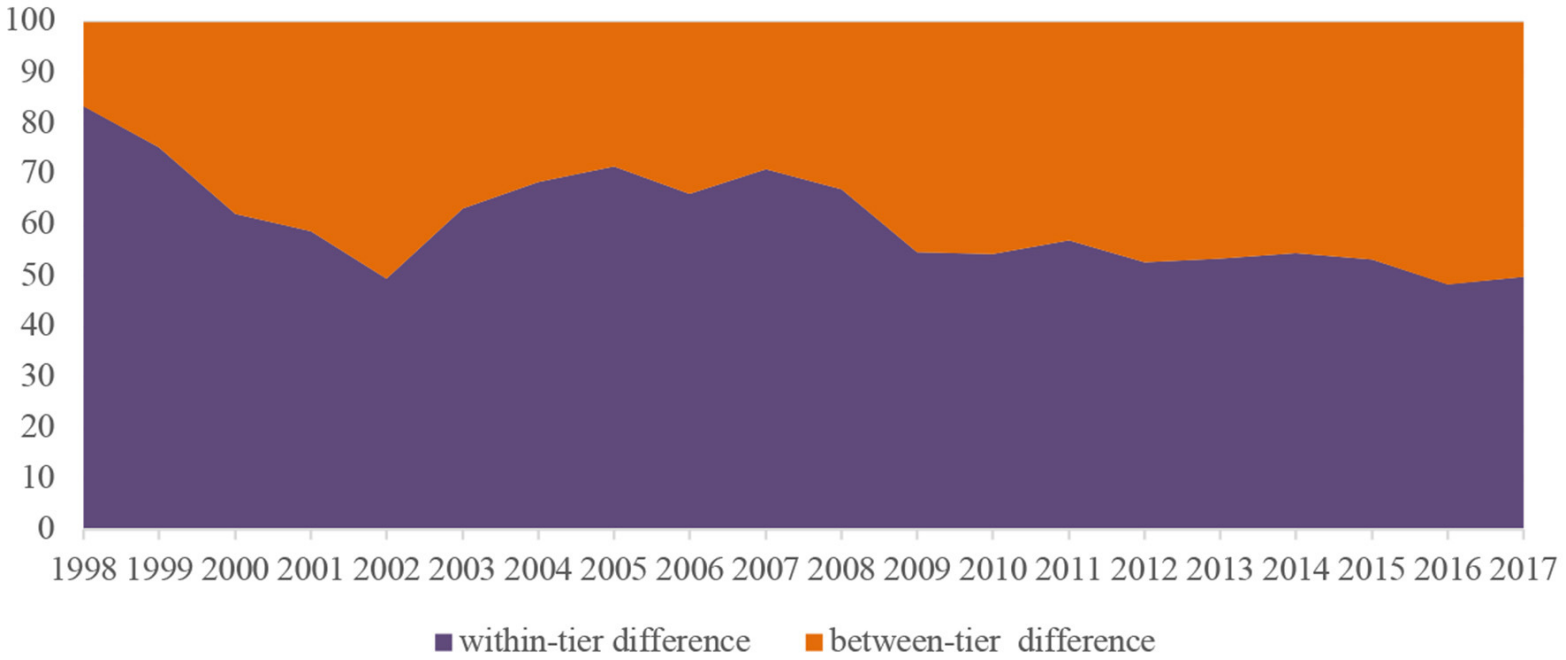
Within-tier and between-tier differences in funding per student among HPIs in China.

Figures 4, 5 display the per student funding differences in HPIs across tiers. As shown in Figure 4, the Gini coefficient of funding per student across all levels of institutions has fluctuated over years ranging from 0 to 0.25, presenting a relatively fair layout in each tier of health professional institutions.

As for the shares of differences within groups and between groups, the difference of per student funding within groups witnessed a general decrease from 83.5% in 1998 to 49.7% in 2017, suggesting the stratification of HPIs has played an increasingly important role in explaining the disparities in the funding per student. Nevertheless, the difference within groups still accounts for a relatively large proportion, which can be attributed to the differences in the scale of institutions within the same tier (Figure 5).

## Discussion

### A Move on the Financing Gaps in Health Professions Education in China

The descriptive statistics in our study showed that the number of HPIs and the average funding per institution of all tiers grew steadily between 1998 and 2017. This was directly related to China's college expansion since 1998. In order to promote economic growth, and achieve national quality improvement, the MOE in China implemented the *Educational Promotion Action Plan for the 21st Century* which rapidly expanded the enrollment in tertiary education (29). Meanwhile, the Chinese government has significantly increased the expenditure on higher education (20). As stated in a series of related regulations such as the Education Law (30), the growth of government funding support for education at all levels should overtake the regular budget of the same level, and the funding per student, as well as the salaries for faculty, should increase gradually. Nevertheless, the rapid expansion in tertiary education in the first few years aggravated the financial burden of governments at all levels, which, regrettably but inevitably, led to the stagnation in the increase of funding per student (31). Although the funding per institution nationwide increased by 17.5 times from 1998 to 2017, the funding per student had only a limited increase of 1.3 times during the period. The basic structure of national higher education was not finalized until around 2008 (30), after which the enrollment numbers have gradually stabilized and the increase in per-student appropriation has gained momentum.

The Chinese government has always attached great importance to the development of the healthcare system and HPE, and have provided with policy guarantee for the year-on-year growth in medical finance. Particularly in the past decade, the Chinese government has established a mechanism of attracting funding through multiple channels in order to support HPE. By 2012, the central government had raised funding per health professional student to $4,000 (¥27,000 Chinese Yuan), reaching the highest standard of students' educational cost in various disciplines (32, 33). In 2014, the MOE pointed out that it was necessary to improve the multi-channel financing mechanism, coordinate the use of resources from all parties including the government, universities, hospitals, and society, and further increase funding in HPE (34). China has gradually formed a diversified pattern of finance on the basis of governmental appropriation and multi-channel funding. Indeed, the growth rate of per-student funding of HPIs in China has overtaken the average level of OECD countries (35).

Despite of the narrowing gaps in the finance of HPE with the developed countries and regions, China still needs to take more measures to further raise funding in HPE and attract more social capital to higher educational institutions. For instance, the average revenue per institution among the 84 fully-accredited public medical schools of the Association of American Medical Colleges (AAMC) in 2017 amounted to $717 million (36), which was almost 3 times more than the 152 public HPIs offering diploma degree in China ($262 million). Taken the small enrollment scale of American medical schools into consideration, the gaps in funding per student may be even greater. It is gratifying that the Chinese government has made continuous and unremitting efforts in the investment of education by regarding the development of education as infrastructure construction, and the funding in education as a basic investment (29). In 2008–2009, the Chinese government shifted its focus from HPIs affiliated with the central government to local health professional institutions (37), which required further increases in both per-student funding and special funds in teaching (31). It has been advocated that the financial needs of HPE should be satisfied through three main sources, i.e., the investment of governments at all levels, the share in educational costs by individuals and social capital, and the support for school-run industries in self-financing. China will gradually establish a financial system for HPE that suits its national conditions as well as the status of health professional institutions.

### Distinct Hierarchy in Health Professional Institutions in China

The results above indicated that the financial differentiation between tiers of HPIs gradually became more accentuated, while the funding gaps contracted within tiers. Concerning the structure of funding sources, the finance system of tertiary education relies mainly on governmental funding, supplemented by multiple channels including tuition, school-run industries, and social donations (20). As for the financing from the competent authorities, the funding of the first-tier universities usually comes from the central government, which is financially more abundant than local governments, especially after the implementation of the tax system reform in 2001. The second-tier universities have two main sources, i.e., the local government finance as well as the special funding from relevant ministries and commissions; and the colleges and universities at the third tier rely heavily on local governments. Considering that the economic development is unbalanced among different provinces and cities, and that local governments may shoulder the financing of multiple institutions, the non-*Project 211* HPIs receive only limited funding support, and their fiscal revenues are quite heterogeneous compared with the upper tiers (38, 39). Apart from governmental finances, tuition fees also play significant roles in the financing of higher educational institutions, and since the public institutions are uniformly priced in China, revenues from tuition fees may be different regarding the scales of institutions. In 2017, governmental funding accounted for 58.2% of the total financing, while tuition fees comprised 22.5%, with the remaining 19.3% from other sources. It should be noted that the financing excluding governmental funding and tuition for the three tiers of institutions accounted for 47.4, 21.6, and 12.9%, respectively, suggesting that it is more difficult for the lower-tier colleges and universities to obtain funds from other channels under the stratified structure. In addition, with regard to the geographic disparity of health education financing, the funds of medical schools in eastern China were significantly higher than the central part, leading to a distinct regional disparity nationwide (35).

The stratification of tertiary education universities in China has increased substantially after the initiation of *Project 211* and *Project 985* at the end of the 20th century. Those selected key universities, with outstanding academic accumulation and profound historical heritage and often located in the political or economic centers of administrative regions, have been obtaining both resource support and policy preferences. In return, the academic strengths and reputation of the upper-tier universities gradually became dominant, which necessarily attracted more non-governmental funding and social donations. Thus, the Matthew effect caused by the stratification of institutions has progressively widened the gap in funding between different tiers of institutions. The *Double First-Class* program with a broader geographical spread in 2015 marked a transition in the competition between institutions from an inherent identity mechanism to a more open competition mechanism. The *Double First-Class* program adopts a rolling elimination system with innovative indicators for evaluation and encourages more institutions to participate in competition and construction. All the colleges and universities should be evaluated every five years, and those which cannot reach the standards of first-class universities and first-class disciplines would be forced out. This, undoubtedly, will reduce the stratification of health professional institutions and strengthen their internal constructions.

### Challenges and Opportunities of Stratified Health Professional Education Worldwide

The analysis in this paper provides an overall picture of finance for HPIs in China, which is of great importance for understanding the challenges and opportunities confronted by HPE worldwide. In fact, the decomposition of funding in the US higher education institutions from 2004 to 2017 also revealed increasing inequality in total expenditures and decreasing inequality in per-student funding (2).The stratified development in HPE has posed a severe challenge to the unified quality assurance for medical personnel training. Health professional institutions of lower tiers are disadvantageous in competitions concerning their scarcity of high-level talents, the lagging of comprehensive education reforms, and the limited capability to attract external resources. This will restrict their further development and progress, and the quality of their student training may also hardly be guaranteed.

Nevertheless, the stratification of HPIs in China can be regarded as intentional action to a certain extent. For developing countries as large as China, it is unrealistic to expect health professional institutions to cultivate students with uniform standards. The academic capabilities are inherently heterogeneous for HPIs of different tiers in different regions, which, definitely, would exert differential impacts on the quality of student output. Therefore, it is of practical significance to first give priority to the development of high-level institutions with limited resources, establish their exemplary role and then facilitate the development of other levels of institutions. The progress of Chinese HPE has been widely acknowledged in the recent decades. According to Academic Ranking of World Universities published by Shanghai Jiao Tong University, 17 institutions in China were shortlisted in the top 500 in 2017, while only 6 were shortlisted in 2005 (40). The guarantee of the quality of personnel training in HPIs can directly promote the improvement of the quality of medical and health services. According to the Lancet ranking of 195 countries and regions in terms of medical quality and accessibility, China achieved a substantial leap with the ranking from the 110th in 1995 to the 48th in 2016. Moreover, China performs significantly better than the average level of middle-income and high-income countries in respect of the main health indicators of residents assured by medical and health services.

The hierarchical development of HPE can be regarded as an effective pathway for developing countries. It may allow certain key universities to take the lead in exploring effective ways of education reform and in establishing pilots for new policies and methodologies. In addition, students from those key universities can be regarded as outstanding talents to meet complicate needs of society. More importantly, the establishment of an open competition mechanism such as the *Double First-Class* program in China can reduce the solidification of the superior status for certain institutions and promote healthy competition by evaluating institutions regularly. In this way, stable development of HPE can be advocated by maintaining the competitive and cooperative relationship between different tiers of colleges and universities.

There were several limitations of this study. First, the paper explored the financing distribution among HPIs, which included not only independent colleges of medicine and pharmacy, but also comprehensive universities with medical-related departments; thus, the funding per institution might be overestimated. Meanwhile, given that students majoring in health professions usually have access to larger shares of financing resources compared to students of other disciplines (32), the per-student funding in our study may be underestimated. In addition, the HPE in our study mainly refers to undergraduate study, failing to take postgraduate study and continuing education into consideration.

## Conclusion

This paper presents an overview of the education finance of HPIs in China over the span (1998–2017), and explores changes in the disparities among different tiers of institutions. The main conclusions are as follows: First, the number of HPIs in China has kept growing over the past two decades, with both the funding per institution and the funding per student increasing steadily. Second, the funding per institution as well as the funding per student in the top-tier institutions maintained to be much higher than those in the lower-tier institutions, while the gap has continued to be widened in recent years. Third, the school funding was more equitably distributed within each tier of institutions, with the Gini coefficient of each tier remaining under 0.3 over the past two decades; yet the share of between-tier difference rising from 29.7% to 77.9%, indicating that the disparity between different tiers become more dominant over years. In particular, the financial resources were more equitably distributed within the top tiers of institutions, while the funding allocation among the lower-tier colleges and universities appeared to be more unfair. In sum, the paper has proposed the need for increasing the per-student funding in China, and emphasized the probability of the stratified development of HPIs for developing countries and regions.

## Data Availability Statement

The raw data supporting the conclusions of this article will be made available by the authors, without undue reservation.

## Author Contributions

HW, WG, and WL conceived the work. WG, WL, and YZ performed data analysis. WG, WL, and YZ were involved in manuscript writing. HW, YZ, and YZh were involved in manuscript revision. WG and WL contributed equally to this work. All authors contributed to the article and approved the submitted version.

## Funding

This research was funded by the National Natural Science Foundation of China for Young Scholars, P.R.C., grant number 71804005.

## Conflict of Interest

The authors declare that the research was conducted in the absence of any commercial or financial relationships that could be construed as a potential conflict of interest.

## Publisher's Note

All claims expressed in this article are solely those of the authors and do not necessarily represent those of their affiliated organizations, or those of the publisher, the editors and the reviewers. Any product that may be evaluated in this article, or claim that may be made by its manufacturer, is not guaranteed or endorsed by the publisher.

## Abbreviations

AAMC: the Association of American Medical Colleges
HPE: Health professional education
HPIs: health professional institutions
MOE: Ministry of Education
NHC: National Health Commission
TCM: traditional Chinese medicine
WDMS: World Directory of Medical Schools
WFME: World Federation for Medical Education.
